# Phosphate Metabolism and Pathophysiology in Parathyroid Disorders and Endocrine Tumors

**DOI:** 10.3390/ijms222312975

**Published:** 2021-11-30

**Authors:** Guido Zavatta, Paola Altieri, Giulia Vandi, Valentina Vicennati, Uberto Pagotto, Fabio Vescini

**Affiliations:** 1 Division of Endocrinology and Diabetes Prevention and Care, IRCCS Azienda Ospedaliero-Universitaria di Bologna, Department of Medical and Surgical Sciences (DIMEC), Alma Mater Studiorum University of Bologna, Via Massarenti 9, 40138 Bologna, Italy; guido.zavatta2@unibo.it (G.Z.); paola.altieri@unibo.it (P.A.); giulia.vandi@studio.unibo.it (G.V.); valentina.vicennati@aosp.bo.it (V.V.); uberto.pagotto@unibo.it (U.P.); 2 Endocrinology and Metabolism Unit, University Hospital Udine, 33100 Udine, Italy

**Keywords:** parathyroid hormone, hyperparathyroidism, phosphate, hypoparathyroidism, osteomalacia, endocrine tumor

## Abstract

The advent of new insights into phosphate metabolism must urge the endocrinologist to rethink the pathophysiology of widespread disorders, such as primary hyperparathyroidism, and also of rarer endocrine metabolic bone diseases, such as hypoparathyroidism and tumor-induced hypophosphatemia. These rare diseases of mineral metabolism have been and will be a precious source of new information about phosphate and other minerals in the coming years. The parathyroid glands, the kidneys, and the intestine are the main organs affecting phosphate levels in the blood and urine. Parathyroid disorders, renal tubule defects, or phosphatonin-producing tumors might be unveiled from alterations of such a simple and inexpensive mineral as serum phosphate. This review will present all these disorders from a ‘phosphate perspective’.

## 1. Introduction

Although the interpretation of serum calcium abnormalities might not be straightforward for the endocrinologist, phosphate metabolism alterations are always challenging. Understanding calcium–phosphate metabolism alterations requires deep knowledge of both bone metabolism and kidney physiology and pathology. For a long time, serum phosphate has been considered to be crucial in the management of kidney disease, playing a major role in vascular calcification in chronic kidney failure. In the past 20 years, the role of phosphate has been profoundly reconsidered since many other molecules have been found to play important roles in phosphate homeostasis, beyond the well-known effect of parathyroid hormone (PTH) or renal function. The advent of new insights into phosphate metabolism must urge the endocrinologist to rethink the pathophysiology of widespread disorders, such as primary hyperparathyroidism, or rarer endocrine disorders known to deeply affect quality of life, such as hypoparathyroidism and tumor-induced osteomalacia (TIO). Rare diseases of mineral metabolism have been and will be a precious source of new information about phosphate and minerals in the coming years. For example, not only the discovery of fibroblast growth factor 23 (FGF-23) [1,2] but also of other small molecules affecting phosphate levels in the blood [3] has promoted great enthusiasm in the endocrinology community involved in mineral metabolism. This review will focus on common diseases managed by endocrinologists, with a special focus on phosphate metabolism based on the most recent available evidence, as of 2021. The review will also briefly deal with phosphatonin-secreting tumors, in which circulating molecules can lead to the well-known TIO syndrome. These topics will be preceded by a brief review of phosphate physiology and by a few practical suggestions on how to quickly interpret phosphate metabolism in everyday clinical practice.

## 2. Overview of Phosphate Physiology and Pathophysiology

While 99% of the total calcium of the human body is located in the bone, by comparison, approximately 85% of phosphate can be found in the skeleton. The remaining phosphate is distributed in other tissues, where this molecule is involved in several essential biological processes. Inorganic phosphate is composed essentially of two ions: dihydrogen phosphate (H_2_PO^4−^) and hydrogen phosphate (HPO_4_^2−^). Only a small fraction of phosphate, approximately 10%, is complexed with cations (calcium or magnesium) or proteins [4].

Intracellular and extracellular phosphate concentrations are maintained constantly by means of sodium–phosphate cotransporters located across the plasma membrane. These cotransporters are subject to endocrine bioregulators, including PTH and FGF-23 within specific organs.

Several biological molecules are made up of organic phosphate: nucleic acids, phospholipids, carbohydrates, adenosine triphosphate (ATP), 2,3-diphosphoglycerate (2,3-DPG), and creatine phosphate, thereby making phosphate a ubiquitous player in the human body, with a special role in the energy compart. In addition, phosphate is involved in intracellular protein phosphorylation, where it serves as one of the main actors to control intracellular signal transduction and, eventually, gene regulation.

Pathophysiology of abnormal serum phosphate is dependent on alterations of intracellular or extracellular phosphorus, or both. In fact, clinical manifestations of low serum phosphate or hypophosphatemia are mainly the result of decreased intracellular phosphorus. Low 2,3-DPG causes hemoglobin to have more affinity to oxygen, preventing the release of oxygen to tissues. ATP production is impaired, thus resulting in impaired cellular function. On the other hand, high serum phosphate or hyperphosphatemia promotes calcium–phosphate deposition, known as calcification, a typically extracellular process often occurring in soft tissues or vessels. Calcium–phosphate deposition becomes pathological [5] when the calcification is able to determine the abnormal function of the tissue or specific symptoms.

Symptoms of hypophosphatemia or hyperphosphatemia might often be nonspecific and easily overlooked. If serum phosphate is low, muscle weakness and fatigue are commonly encountered. Signs of rickets or osteomalacia can also be present. More severe clinical consequences might occur if hypophosphatemia development is acute and severe, thereby leading to respiratory dysfunction, hemolysis, rhabdomyolysis, or even cardiac failure. By comparison, symptoms of chronic hyperphosphatemia are more subtle and often nonspecific, while in acute hyperphosphatemia symptoms of hypocalcemia (tingling, paresthesias, and muscle cramps) are the main clinical presentation. Chronic hyperphosphatemia might be evident with palpable, hard, subcutaneous nodules, consistent with soft tissue calcifications.

Serum phosphate is significantly higher in younger patients (e.g., infants and children) compared to adults, and abnormalities of phosphate values can be missed if the adult reference range is used to evaluate pediatric patients. Therefore, it is important to recognize hypophosphatemia when serum phosphate concentrations are below the lower limit of normal for a given age. The opposite applies to hyperphosphatemia. This biochemical feature can be explained by a positive phosphate balance in growing children; phosphate absorption in the small intestine is greater than renal phosphate excretion to allow skeletal accrual [4]. In contrast, in adults, the phosphate balance is equal to zero unless parathyroid disorders or other causes are present.

**Figure 1:**
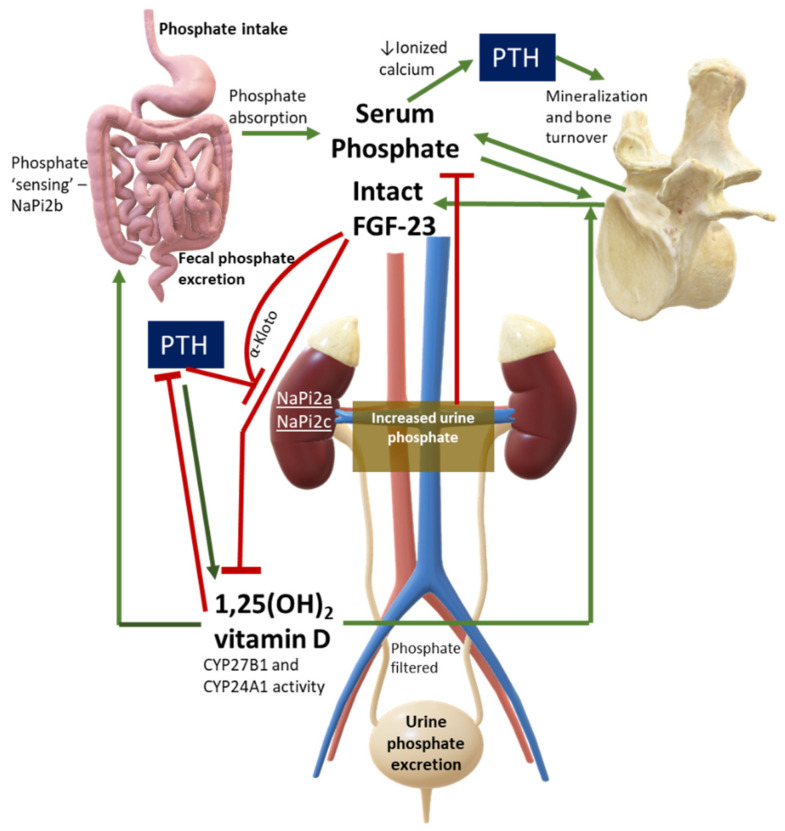
Phosphate Physiology. Green lines are stimulatory pathways, while red lines stand for inhibitory pathways. This figure can also be used to interpret diseases with PTH or FGF-23 excess or deficiency, or in case of abnormalities in phosphate cotransporters. Legend: CYP27B1, 1-alpha hydroxylase; CYP24A1, vitamin D 24-hydroxylase; NaPi, sodium-phosphate cotransporter.

Serum phosphate must not be evaluated alone but should always be assessed under a pathophysiological perspective. Major hormones involved in phosphate physiology include 1,25(OH)_2_ vitamin D, parathyroid hormone (PTH), and phosphaturic peptides such as FGF-23. Figure 1 shows a simplification of normal phosphate physiology.

Hypophosphatemia is a common biochemical abnormality in hospitalized patients and has been associated with poor outcomes [6]. Low serum phosphorus is seen in up to 5% of hospitalized patients [7,8]. Phosphorus is largely (60–65%) and easily absorbed in the small intestine, and its absorption is mediated by the sodium-dependent phosphate cotransporter type IIb (NaPiIIb). The higher the dietary load, the higher the intestinal absorption, with a resultant higher demand for phosphate excretion through the kidneys [9]. A recent study has assessed the biochemical response to inorganic phosphorus in healthy subjects. The study found that acute administration of phosphorus was able to increase postprandial serum phosphate levels and to stimulate the release of the parathyroid hormone, in spite of unchanged concentrations of FGF-23 in up to eight hours of observation [10]. These findings suggested that FGF-23 does not rapidly contribute to phosphate homeostasis, with PTH playing a dominant role in acute circumstances. In other studies, FGF-23 increased in response to phosphate loading, but this was observed only after several days [10].

**Figure 2:**
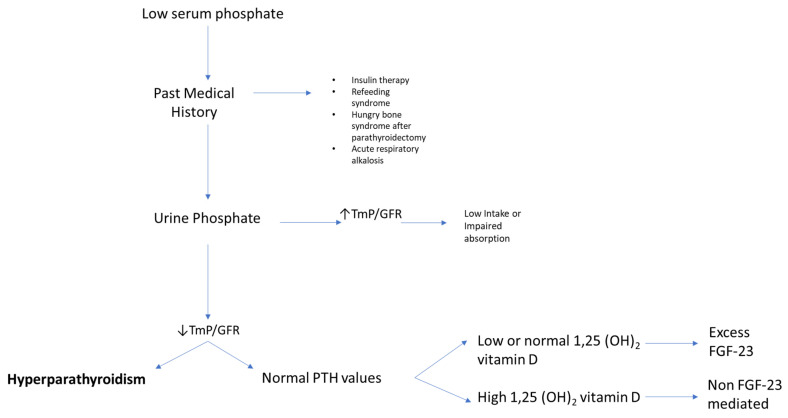
Flowchart for evaluating patients presenting with low serum phosphate (hypophosphatemia). High TmP/GFR is indicative of high renal reabsorption of phosphate, which is appropriate in case of low intake or malabsorption. When TmP/GFR is inappropriately low in the setting of hypophosphatemia, this suggests renal phosphate wasting. Legend: TmP/GFR, maximum tubular reabsorption of phosphate corrected for glomerular filtration rate.

For the clinician approaching phosphate alterations, it is important to know that approximately 80% of filtered phosphate is reabsorbed in the proximal tubule and <10% in the distal segments of the nephron [11]. Renal reabsorption of phosphate progressively increases as the filter load increases, with filtered phosphate reaching at some point a maximum tubular reabsorption rate or TmP. Because TmP depends on renal function, the correct parameter to estimate phosphate renal tubular reabsorption is TmP/GFR. If TmP/GFR is low despite low serum phosphate, this means that the kidney is wasting phosphate. This ratio should be calculated from fasting serum phosphate and creatinine and a second void urine sample. In patients with hypophosphatemia, fractional excretion of phosphate (FEP) may also be useful. FEP can be calculated from the following formula: FEP = (Urinary Phosphate × Plasma Creatinine)/(Plasma Phosphate × Urinary Creatinine). When low serum phosphorus is diagnosed, a rule of thumb is that FEP > 5% or 24-h Urinary Phosphate > 100 mg; both can be used to confirm increased renal tubular phosphate loss [5]. A diagnostic flowchart for hypophosphatemia is shown in Figure 2 to guide classification of the underlying disorder. Of note, intestinal malabsorption of phosphorus, phosphorus redistribution into cells, and increased loss through the kidney are the three most common mechanisms behind hypophosphatemia.

## 3. Why Measure Serum Phosphate?

Low mineralization of the skeleton or ectopic calcification are opposite examples of how derangements of phosphate physiology might easily affect human tissue integrity. Serum phosphate abnormalities almost always hide an underlying health issue. The next few paragraphs will aim to present the importance of phosphate levels in parathyroid disorders, both in primary hyperparathyroidism and in hypoparathyroidism. Tumor-induced osteomalacia will then be discussed with a focus on phosphate and PTH interactions.

## 4. Phosphate in Primary Hyperparathyroidism

An early study from Columbia University [12] investigated the interactions between oral phosphate and bone health indices, including parathyroid hormone levels. Short term oral phosphate administration was shown to decrease serum calcium levels and raise PTH levels within hours, and to subsequently cause a stable rise of PTH from the third day. The presumed mechanism was attributed to decreased ionized calcium, leading to increased PTH secretion. The results of this study, conducted in 1986, are still current. Calcium and phosphate relationships have been recently reappraised in patients with primary hyperparathyroidism, with more studies focusing on ratios between minerals rather than the single mineral alone. Nowadays, it is essential to recognize early or milder phenotypes of primary hyperparathyroidism, e.g., normocalcemic primary hyperparathyroidism (NHPT), where, by definition, only the parathyroid hormone is altered, and the measurement of serum calcium alone probably becomes less useful because it is persistently within normal limits. Adding serum phosphate to the diagnostic flowchart of primary hyperparathyroidism could confirm or exclude an NHPT diagnosis, at least according to some studies [13,14].

In traditional primary hyperparathyroidism, serum phosphate is usually in the lower range of normal. In about one-quarter of patients, it is frankly below normal. It is unusual to find patients with primary hyperparathyroidism and serum phosphate values above 3.5 mg/dL in the absence of significant renal insufficiency [15]. The calcium/phosphate ratio might be used as a new index to suspect or confirm a diagnosis of PHPT [13,14]. Madeo and colleagues proposed the specific cut-point for the serum Ca/P ratio of 3.5 when Ca and P were measured in mg/dL [13]. This index had a sensitivity of 89% and specificity of 91% for detecting patients with both classical and normocalcemic primary hyperparathyroidism. In normocalcemic patients, the sensitivity of the Ca/P ratio was lower at 67%, although with comparable specificity. The authors concluded that the Ca/P ratio could be used in primary care settings or during high-volume screenings of patients because it could avoid the unnecessary measurement of PTH in many patients. However, a significant proportion of patients with NHPT might easily be missed if this ratio were used as a screening tool. This is probably due to a milder phenotype of normocalcemic hyperparathyroidism, where serum phosphate values are usually greater compared with traditional PHPT [16]. It is noteworthy that the Ca/P ratio had very high negative predictive values of 88–95%, suggesting that it could be a more reliable tool to exclude rather than confirm milder phenotypes of PHPT.

Another index, the *PFindex*, proposed by Guo and colleagues [17], is instead calculated from (serum calcium × PTH)/serum phosphate, with calcium and phosphate reported in mmol/L, and PTH in pg/mL. These authors retrospectively evaluated 128 patients, either with PHPT or normocalcemic PHPT, undergoing parathyroid surgery. A PFindex >34 was able to discriminate primary hyperparathyroidism from vitamin D-deficient secondary hyperparathyroidism with a sensitivity of 96.9% and a specificity of 97.6%.

Other tools such as Chloride to Phosphate Ratio (Cl:PO_4_ ratio) seem less useful as diagnostic laboratory tests in mild PHPT. A recent study evaluated 226 patients undergoing parathyroidectomy. Of these, 166 patients had serum calcium less than 10.4 mg/dL and were hyperchloremic (serum chloride > 106 mmol/L). Although intriguing, the sensitivity and specificity of the Cl:PO_4_ ratio were 58.4% and 28.6%, respectively [18].

Studies on the clinical role of FGF-23 in PHPT are scarce, although this hormone might have a potential role in the severity of clinical manifestations or symptoms in these patients. Twenty-nine PHPT patients were evaluated prospectively before and after parathyroidectomy [19]. The authors found that 1.25(OH)_2_ vitamin D levels correlated with FGF-23 levels both preoperatively and postoperatively, although they could not find any other significant relationship with calcium, phosphate, and PTH. Another recent study has assessed FGF-23 in PHPT patients [20]. Seventeen hypercalcemic PHPT patients were evaluated before parathyroidectomy and 6 months after surgery. Before surgery FGF-23 levels were higher compared with nine age-matched controls. FGF-23 levels decreased significantly after parathyroidectomy but remained higher compared with controls. These data suggest that FGF-23 is abnormal in PHPT, but its clinical significance and implications deserve further study.

If the interpretation of abnormalities in phosphate metabolism in normal circumstances is challenging, it could be even more challenging in patients with PHPT, which is the third most common endocrine disorder [21]. In fact, underlying phosphate disturbances may be missed in PHPT. For example, a study from the Mayo Clinic [22] aimed to characterize a group of 50 patients with coexisting PHPT and sarcoidosis, which can both present with hypercalcemia. The study found that mean serum phosphate levels were 3.3 ± 0.6 mg/dL in this cohort, and that patients with active sarcoidosis had higher serum Angiotensin Converting Enzyme (ACE) levels, lower PTH levels (60 ± 24 vs. 96 ± 41 pg/mL), and lower phosphate levels (2.7 ± 0.6 vs. 3.2 ± 0.5 mg/dL), as compared with patients with inactive sarcoidosis. Of note, serum phosphate levels were unexpectedly lower in patients with active sarcoidosis. The authors concluded that there could be other mechanisms causing lower serum phosphate in this population.

A very recent study rediscovered a new pathogenetic role for phosphate in primary hyperparathyroidism [23]. Castellano et al. evaluated a series of 472 consecutive patients with PHPT and aimed to establish a relationship between phosphate levels and clinical manifestations. These authors found that 41.9% of the patients presented with low serum phosphate mildly decreased (between 2 and 2.5 mg/dL) in the vast majority of cases (84.9%). Patients with a more pronounced phenotype of PHPT had a higher prevalence of hypophosphatemia and nephrolithiasis, but not osteoporosis. The authors suggested that moderate hypophosphatemia (1–1.9 mg/dL) in PHPT should be considered as a supportive, inexpensive, and easily available means to identify asymptomatic PHPT patients who may benefit from surgery. Cinacalcet, compared to placebo, can also increase serum phosphate levels in patients with PHPT [24], but its impact on mineralization and bone density is probably neutral in these patients [25].

## 5. Phosphate in Hypoparathyroidism

Phosphate in hypoparathyroidism is by, definition, high or high-normal because of insufficient renal excretion caused by the absolute or relative absence of PTH. Phosphate abnormalities, which are not easily corrected by conventional therapy, are thought as one of the pathogenetic factors for some complications of hypoparathyroidism, such as calcification in renal and extra-renal tissues [26]. Conventional therapy for hypoparathyroidism aims to maintain serum phosphate in the normal range and keep calcium–phosphate product below the target of 55 mg^2^/dL^2^. Newer therapies, based on PTH analogues, by contrast, are able to restore normal phosphate excretion in a few days, thus avoiding hyperphosphatemia. Recombinant human PTH (1-84) (rhPTH1-84) was shown to reduce serum phosphate levels and to improve calcium–phosphate product while maintaining 1,25(OH)_2_ vitamin D and serum calcium in the normal range, along with a reduction in calcium and active vitamin D supplements [27,28]. A recent phase two trial on TransCon PTH has shown promising results regarding phosphate control in adult hypoparathyroidism [29].

FGF-23 is elevated by chronic hyperphosphatemia [30]. A very recent and well-conducted clinical study [31] has shed light on the interdependent physiology of PTH and FGF-23 in patients with hypoparathyroidism. Since both FGF-23 and PTH are phosphaturic hormones, it was anticipated that patients with hypoparathyroidism, treated with synthetic human PTH (1-34), would serve as an optimal model to clarify which of either hormone would have a prominent role over the other in determining phosphate excretion. Patients on conventional treatment were tested immediately before and up to 24 h after rhPTH (1-34) administration. Four hours after rhPTH (1-34), nephrogenic cAMP increased significantly, and this was accompanied by a significant reduction in tubular reabsorption of phosphate (TRP). Serum phosphate at 4 h decreased also. Intact FGF-23 was elevated at the baseline and trended downward over the 24-h period until it was significantly lower than the baseline. Calcium and 1,25(OH)_2_ vitamin D did not change over 24 h. Therefore, the study demonstrated that PTH administration overcame FGF-23 resistance, suggesting that the full phosphaturic effect of FGF-23 might be PTH-dependent. The study clearly showed that PTH and FGF-23 act interdependently to maintain phosphate homeostasis. To support the hypothesis of ‘PTH-FGF-23 interdependency’, the authors evaluated one patient with Hyperphosphatemic Familial Tumoral Calcinosis, which is a rare genetic condition of functional FGF-23 deficiency, characterized by hyperphosphatemia, elevated 1,25(OH)_2_ vitamin D, and increased phosphate reabsorption. In this patient, administration of rhPTH (1-34) was not able to bring about a phosphaturic effect. This study provided experimental data in vivo that both FGF-23 and PTH are needed to regulate normal phosphate reabsorption in the renal tubule. It is, however, unknown whether acute and severe hyperphosphatemia might cause a much greater FGF-23 release in order to overcome the FGF-23 resistance described in this study. In fact, the results of this study apply to biochemically well-controlled patients with chronic hypoparathyroidism with normal kidney function. In other words, it might be that higher FGF-23 levels are required to produce phosphate wasting in patients with hypoparathyroidism.

Soft tissue calcification has been associated with abnormalities of both calcium and phosphate for a long time [32]. Similar to PHPT, serum calcium/phosphate ratio might also be a valuable index to evaluate the onset of some complications in hypoparathyroidism, such as soft tissue calcifications. Basal ganglia calcifications were shown to be linked to a reduced time-weighted average of serum calcium and calcium/phosphate ratio prior to the onset of brain calcification, as opposed to calcium x phosphate product, which was not associated with this complication [33]. However, a high calcium × phosphate product was historically evaluated in renal replacement therapy patients to set a threshold above which vascular and visceral calcifications were likely to develop. The cut-off point was initially set at 60 mg^2^/dL^2^, but it was reduced to 55 mg^2^/dL^2^ in 2000 because mitral valve calcification was still observed between these values. This could suggest that patients with optimal biochemical control of the disease might not be at risk for vascular calcification, but are still at risk for basal ganglia calcification if their average calcium/phosphate ratio is kept too low, likely due to frequent episodes of hypocalcemia. Further evidence from clinical trials evaluating PTH analogues could help clarify whether inverting the calcium/phosphate ratio could help prevent or reduce the appearance of this complication.

Regarding renal calcification or nephrolithiasis, it is still unknown which among conventional therapy, hypercalciuria, calcitriol excess, or phosphate control could have a major influence on the onset of these complications. Phosphate in the urine of patients with hypoparathyroidism is lower than that seen in the general population. According to recent studies, excessive calcium excretion might have a dominant role compared to phosphate metabolism, although uncertainty remains about this pathogenetic hypothesis [34].

Regarding ectopic calcification, it is important to note that the biochemical reactions behind this process appear to be much more complex than what can be expressed by a simple index, such as the serum calcium x phosphate product [35]. Other chemical mediators likely play a significant role, with pyrophosphate levels possibly modulating calcium deposition [36]. Circulating pyrophosphate, a natural inhibitor of hydroxyapatite formation, is usually removed by phosphatases. Therefore, alterations in pyrophosphate metabolism may also be linked to calcification, for example, vascular calcification [37]. The calcification process in hypoparathyroidism deserves further study.

## 6. Phosphate in Paraneoplastic Endocrine Tumors

### 6.1. Tumor-Induced Osteomalacia

#### 6.1.1. Diagnosis

Tumors may produce factors called phosphatonins which are able to cause phosphate wasting and are also capable of decreasing the production of 1,25 (OH)_2_ vitamin D. These disorders are called a unique name: tumor-induced osteomalacia (TIO) or oncogenic osteomalacia [5]. It is a rare acquired syndrome, first described in 1947 [38], in which tumors oversecrete FGF-23, MEPE, sFRP4, FGF-7, and probably many other, yet unknown, phosphatonins. Long-standing bone, joint, and muscle pain with fractures and fatigue, along with elevated total and bone-specific alkaline phosphatase plus hypophosphatemia are suspicious for TIO. In adult patients with this clinical phenotype, TIO must be ruled out because genetic hypophosphatemia is highly unlikely unless they have autosomal dominant hypophosphatemic rickets (ADHR). Table 1 summarizes the main causes of hypophosphatemia due to phosphate wasting from the kidneys, including genetic etiologies. Clinicians should bear in mind that genetic disease is rarely the case in the absence of clinical features, such as bowed legs and short stature, which are classical signs of early onset of rickets.

#### 6.1.2. Clinical Presentation

The most frequent clinical presentation of TIO is in adult patients (>50 years), although the age of diagnosis and onset may vary. Since tumors are usually small-sized, the time to localize and resect these lesions can be as long as 5 years or more [39]. Tumors causing hypophosphatemia are called phosphaturic mesenchymal tumors [40], occurring either in the bone or in soft tissues. FGF-23 levels are often increased in these patients [41] and usually decrease after complete resection of the tumor [42].

These tumors may grow in the distal extremities, nasopharynx, sinuses or may also be subcutaneous. A Dotatate-PET scan can detect the tumor [43], as these lesions often express somatostatin receptors. Complete resection of the tumor allows osteomalacia to resolve, with normalization of FGF-23, 1,25(OH)_2_ vitamin D, and other minerals. Resection can be performed through surgery, but radiofrequency and cryoablation have also been reported as successful solutions [5]. Until the tumor is not localized, oral phosphate and calcitriol should be given to aid mineralization in the bone and alleviate patient symptoms, and to increase serum phosphate levels. Phosphate (1–2 g/day) and calcitriol (1–3 mcg/day) are given in multiple daily doses, and, with long-term exposure, patients may develop secondary or tertiary hyperparathyroidism [44]. The presumed mechanism is likely due to both deficient 1,25(OH)_2_ vitamin D production and phosphate supplementation, which lowers ionized calcium, thereby continuously triggering PTH release. A careful balance between phosphate supplementation and calcitriol is necessary. These patients can sometimes benefit from parathyroidectomy or cinacalcet [45] to control high PTH levels, which fuel phosphate wasting, thereby increasing the need for phosphate supplements.

The driving cause of hyperparathyroidism in TIO is probably due to decreased ionized calcium and the underlying inhibition of 1,25(OH)_2_ vitamin D production mediated by FGF-23 excess. These pathogenetic mechanisms result in parathyroid tissue proliferation, with the subsequent possible development of parathyroid gland autonomy. Disruption of the physiological regulation of PTH secretion may contribute to hyperparathyroidism and several case reports have been published in patients with X-linked hypophosphatemia [46]. In an observational study from France evaluating 70 adult patients with X-linked hypophosphatemia (XLH) [46], hypercalcemic hyperparathyroidism was present in 7 of them, thus suggesting that deficient production of 1,25(OH)_2_ vitamin D may result in the development of primary hyperparathyroidism. However, there were no differences in 1,25(OH)_2_ vitamin D levels between patients with PHPT and patients without PHPT, perhaps due to concomitant inhibitory action of FGF-23 excess and stimulatory action of PTH excess on 1α-hydroxylase.

The anti-FGF-23 antibody, burosumab, was approved on 18 June 2020 by the FDA for the treatment of TIO following two phase two studies [47,48]. Burosumab is also approved for the treatment of pediatric and adult X-linked hypophosphatemia. This new therapeutic solution is able to normalize phosphate levels if the tumor is not resectable or not yet localized, or relapses after surgery. Burosumab binds to circulating intact FGF-23, thus preventing its action on the FGFR1 receptor on the renal tubule, eventually leading to increased phosphate reabsorption and normal serum phosphate values.

### 6.2. Other Solid Tumors and Phosphate

Hypercalcemia of malignancy (HCM) is a common complication of advanced cancer. Although it is not within the scope of this review, interpretation of calcium–phosphate laboratory values can sometimes be challenging in patients with metastatic tumors. A recent retrospective chart review study [49] found that patients with HCM with 1,25(OH)_2_ vitamin D elevation responded less favorably to antiresorptive treatment. A negative correlation was found between calcitriol and serum phosphate levels (Pearson r = −0.261) and between PTH-related peptide (PTHrp) and serum phosphate (Pearson r = −0.4) only in patients without calcitriol elevation, suggesting that assessment of 1,25(OH)_2_ vitamin D in these patients might have clinical significance. Therefore, a higher serum phosphate might suggest a concomitant underlying calcitriol elevation, with implications for treatment choice. However, the authors of this study noted that hypophosphatemia likely was not the driver of calcitriol elevation because the negative correlation was found only in patients without calcitriol elevation. The mechanisms by which calcitriol is produced, therefore, remain unclear, but an assessment of 1,25(OH)_2_ vitamin D could predict its response to certain treatments.

## 7. Conclusions

Phosphate physiology is complex, and interpretation of phosphate alterations in the setting of parathyroid–kidney disorders, endocrine or advanced tumors is even more challenging. Here are a few take-home messages:

- In primary hyperparathyroidism, decreased serum phosphate might be associated with a more severe disease. Patients presenting with moderate hypophosphatemia could benefit from a surgical approach, once other underlying causes of hypophosphatemia have been addressed and excluded;
- In patients with hypoparathyroidism, serum phosphate is associated with soft tissue calcification, although evidence of this is still scarce, suggesting that both calcium and phosphate derangements contribute to this complication. Newer treatments based on PTH analogues used as adjunctive therapies are able to quickly restore normal serum phosphate;
- FGF-23 or other phosphatonin-producing tumors cause a rare paraneoplastic syndrome, TIO, which might easily go unrecognized for a long time despite severe, debilitating symptoms. Awareness of this disorder should be enhanced across the medical community to allow prompt endocrinological referral in case of acquired and unexplained hypophosphatemia.

## Figures and Tables

**Table 1:** Hypophosphatemia due to renal phosphate loss other than primary hyperparathyroidism.

	Disease	Etiology	Pathogenesis
**Hormone Excess**			
FGF-23			
	Tumor-Induced Osteomalacia, TIO	Mesenchimal tumor	Paraneoplastic
	X-linked hypophosphatemia, XLH	PHEX mutation	Inappropriately high production of FGF-23
	Autosomal dominant hypophosphatemic rickets, ADHR	FGF-23 mutation	The mutation makes FGF-23 resistant to cleavage
	Autosomal recessive hypophosphatemic rickets type1, ARHR1	Loss of DMP1	Impaired osteocyte differentiation
	Autosomal recessive hypophosphatemic rickets type2, ARHR2	ENPP1 mutation	Increased FGF-23 production
	Fibrous displasia	GNAS mutation	Dysplastic bone produces excess FGF-23
	Linear nevus sebaceus syndrome	Excess FGF-23 prodution	Dysplastic bone or nevi produce excess FGF-23
	Osteoglophonica dysplasia	FGFR1 mutation	Dysplastic bone produces excess FGF-23
sFRP4	TIO	Mesenchimal tumor	Paraneoplastic
MEPE	TIO	Mesenchimal tumor	Paraneoplastic
FGF-7	TIO	Mesenchimal tumor	Paraneoplastic
**Transporter mutation**			
	Nephrolithiasis/Osteoporosis, Hypophosphatemic, 1 (NPHLOP1)	Heterozygous mutation in the *SLC34A1* gene	Renal phosphate wasting, loss-of-function of NaPi-IIa.
	Nephrolithiasis/Osteoporosis, Hypophosphatemic, 2 (NPHLOP2)	Heterozygous mutation in the *SLC9A3R1* gene	NHERF1 ^1^ mutation and higher responsiveness to PTH, through cAMP production
	Fanconi Renotubular Syndrome 2 (FRTS2)	Homozygous mutation in the *SLC34A1* gene	Loss-of-function of NaPiIIa. Hypercalciuria due to increased serum 1,25-dihydroxyvitamin D levels and increased intestinal calcium absorption
	Hypophosphatemic Rickets and Hyperparathyroidism	Alpha-Klotho translocation	Increased Klotho and FGF-23
	Hypophosphatemic Rickets with Hypercalciuria (HHRH)	Homozygous or compound heterozygous mutation in the sodium-phosphate cotransporter gene *SLC34A3*	Loss of function of NaPiIIc Hypercalciuria due to increased serum 1,25-dihydroxyvitamin D levels and increased intestinal calcium absorption

^1^ Sodium/Hydrogen Exchanger Regulatory Factor 1.

## Data Availability

Not applicable.
